# An update of advanced nanoplatforms for Glioblastoma Multiforme Management

**DOI:** 10.17179/excli2021-4393

**Published:** 2021-11-15

**Authors:** Mariana Amaral, Nuno Cruz, Ana Rosa, Beatriz Nogueira, Diana Costa, Francisco Santos, Mariana Brazão, Pedro Policarpo, Rita Mateus, Yan Kobozev, Catarina Pinto Reis

**Affiliations:** 1 iMED.ULisboa, Research Institute for Medicines, Faculty of Pharmacy, Universidade de Lisboa, Av. Prof. Gama Pinto, 1649-003 Lisboa, Portugal; 2 Faculty of Pharmacy, Universidade de Lisboa, Av. Prof. Gama Pinto, 1649-003 Lisboa, Portugal; 3 IBEB, Biophysics and Biomedical Engineering, Faculty of Sciences, Universidade de Lisboa, Campo Grande, 1749-016, Lisboa, Portugal

**Keywords:** Glioblastoma multiforme, nanosystems, superparamagnetic nanoparticles, polymeric nanoparticles, liposomes, gold nanoparticles

## Abstract

Glioblastoma multiforme (GBM) is a very aggressive and heterogeneous glioma. Currently, GBM is treated with a combination of surgery, radiotherapy, chemotherapy (e.g. temozolamide) and Tumour Treating Fields. Unfortunately, the mean survival is still around 15 months. This poor prognosis is associated with therapy resistance, tumor recurrence, and limited delivery of drugs due to the blood-brain barrier nature. Nanomedicine, the application of nanotechnology to medicine, has revolutionized many health fields, specifically cancer diagnosis and treatment. This review explores the particularities of different nanosystems (i.e., superparamagnetic, polymeric and gold nanoparticles, and liposomes) as well as how they can be applied to the treatment and diagnosis of GBM. As described, the most of the cited examples are on the preclinical phase; however, positive results were obtained and thus, the distance to achieve an effective treatment is shorter every day.

## Introduction

### Glioblastoma

Glioblastoma multiforme (GBM) is a malignant brain tumor which is thought to emerge from neuroglial progenitor cells and neural stem cells (Canoll and Goldman, 2008[20]), being the most common and aggressive type of brain tumor, with a median survival time of 15 months (Stupp et al., 2009[112]). The 2016 WHO classification of central nervous system (CNS) tumors describes it as a grade IV glioma (Louis et al., 2016[75]), with an average incidence of 3.2 per 100.000 persons. These numbers make up 54 % of all gliomas and 16 % of all brain tumors (Tamimi and Juweid, 2017[116]). GBM is a very heterogenic disease, with several different biomolecular markers that predict treatment response: methylation status of the gene promoter for O6-methylguanine-DNA methyltransferase (MGMT) and isocitrate dehydrogenase enzyme 1/2 (IDH1/2) mutation are common in *de novo* GBM, and indicate a form of GBM more responsive to treatment (Thakkar et al., 2014[117]).

Symptoms include increased intracranial pressure, headaches, neurological deficits, seizures and others, depending on the location of the tumor (Alexander and Cloughesy, 2017[4]). Current standard of care is surgery for maximal tumor extraction, with concomitant radiotherapy (RT) and temozolamide (TMZ), and recently Tumour Treating Fields (TTF) (Fernandes et al., 2017[41]; Geraldo et al., 2019[46]). Unfortunately, all of these treatments present a poor or limited outcome, mainly due: to the difficulty of achieving a complete resection of the tumors, the limited delivery of therapeutics across the blood-brain barrier (BBB) and the ability of glioma stem cells to both create and expand GBM populations, and, ultimately, to develop therapy resistance, resulting in tumor recurrence (Marekova et al., 2020[79]). To help overcome the difficulties in treatment of such a lethal disease, fields such as nanomedicine have been explored.

### Nanomedicine

Nanomedicine has become one of the 21^st ^century key sciences, and originated from the development of ultramicroscopic systems (drug carriers, medical devices, etc.), and which allowed for the study of cellular, molecular and atom sized structures in biology, chemistry, and physics (Krukemeyer et al., 2015[65]).

**Figure 1:**
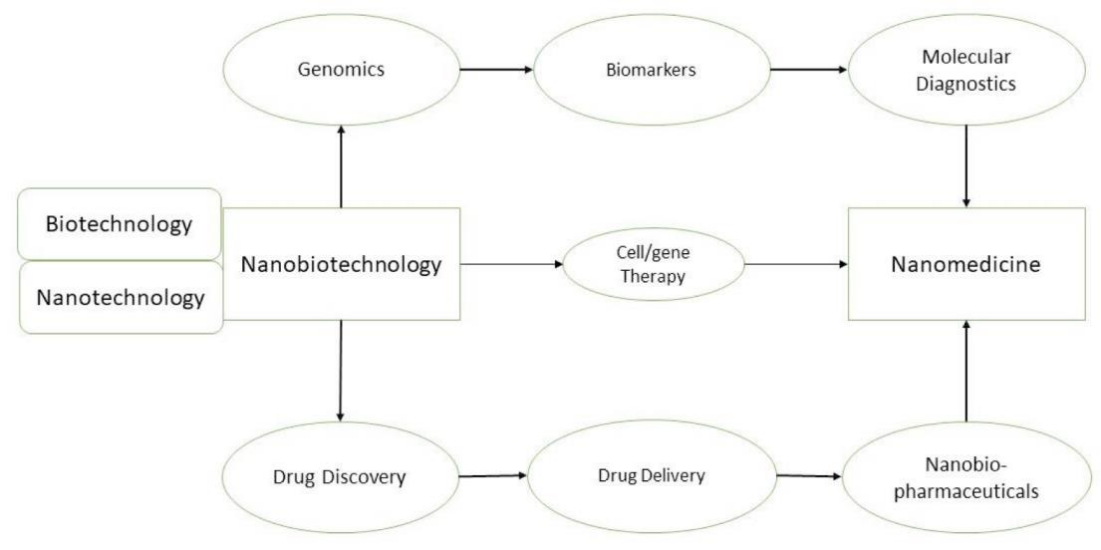
The different relations between Nanobiotechnology and Nanomedicine, and how they are intimately connected.

This nanotechnological approach was the driving force that allowed nanomedicine to establish itself as a fundamental section in science and medicine. Nanotechnology developed rapidly since the beginning, driven by the tremendous progress in the development of new techniques. Nanobiotechnology is concerned with molecular intra- and intercellular processes and, consequentially, is of great importance for nanotechnology applications in medicine. This is definitely apparent in the relation between relevant medical nanotechnologies and possible uses of nanobiotechnology in medicine, as we can see in Figure 1(Fig. 1).

The main uses of nanotechnology in medicine are based on three core concepts (Krukemeyer et al., 2015[65]):

1. Knowledge of molecular medicine in the fields of genetics and synthetically produced or modified microorganisms.

2. Nanomaterials and nanodevices that can be used as biosensors, as transporters of active substances and aides in treatment.

3. Nanotechnologies that can speed up the process of diagnostics and therapy, for tissue repair, as well as the improvement of normal physiological functions. 

## Superparamagnetic Nanoparticles

Superparamagnetic nanoparticles (NPs) can be defined as NPs that present magnetic properties while affected by an external field, thus it renders an opportunity to target specific sites for therapeutic deliver. Nowadays, the approved use of superparamagnetic particles is mostly related to diagnosis. 

In this field, superparamagnetic Iron Oxide NPs (SPIONs) are not only relatively cheap but the most widely used particles in diagnosis and have been researched as a drug delivery system. The core of these particles is usually formed by iron oxide compounds such as a-Fe_2_O_3_ (hematite), c-Fe_2_O_3_ (maghemite) or Fe_3_O_4_ (magnetite) (Avval et al., 2020[11]). These particles can be further modified to achieve specific characteristics and properties for drug delivery, hyperthermia and gene delivery (Marekova et al., 2020[79]).

### Production 

**Figure 2:**
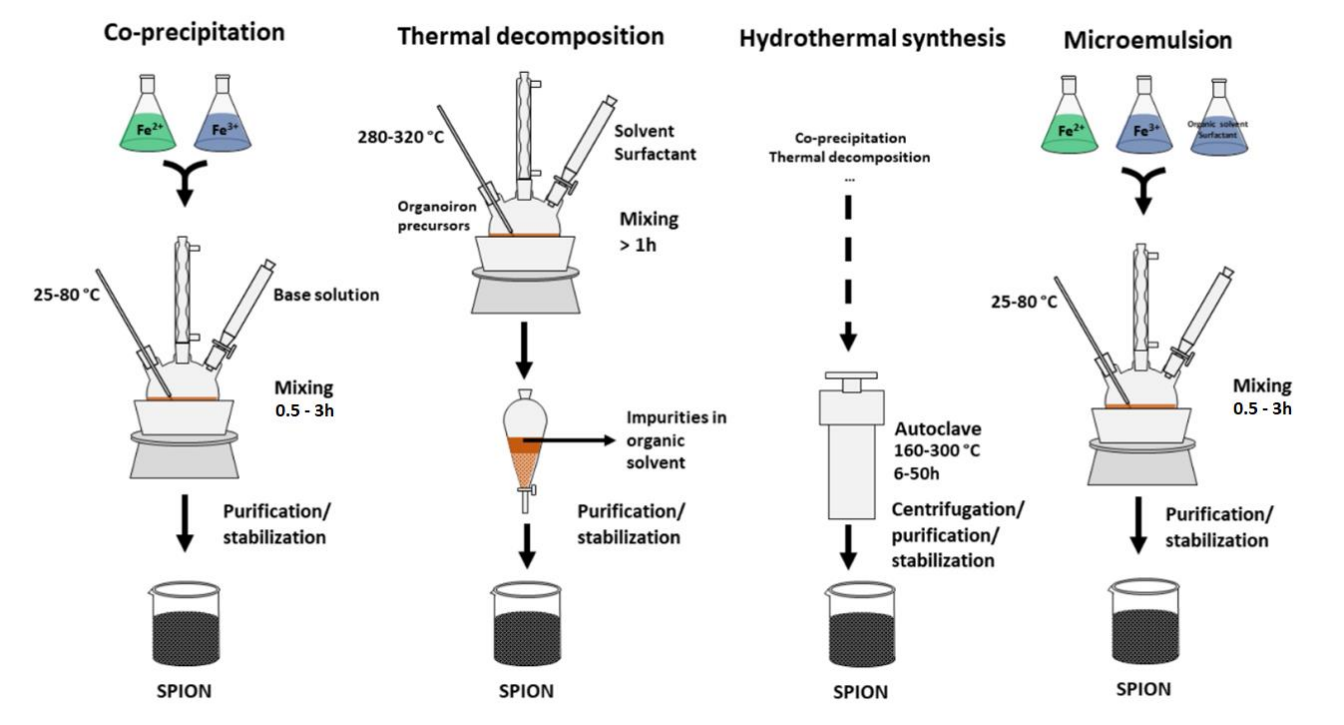
Schematic representation of co-precipitation, thermal decomposition, hydrothermal synthesis and microemulsion methods.

There are many techniques that can be used as strategies for production of SPIONs - the most used methods are co-precipitation, thermal decomposition, hydrothermal synthesis, microemulsion and sol-gel (Figure 2(Fig. 2)). The choosing of a production method depends on the desired characteristics.

### Characterization

The development of medicinal products based on NPs requires standardization of methods to characterize the properties of formulations for such innovative products. American (USP) and European pharmacopeias (Ph. Eur) describe some of the methods which are not directly intended for nanoproducts but can be used for the characterization of NPs. Other methods are also standardized by ISO and ASTM International (Halamoda-Kenzaoui et al., 2019[58]).

Dynamic light scattering (DLS) is a tech-nique widely used for size characterization of NPs in general and is one of the preferred methods for magnetic NPs. Other methods such as transmission electron microscopy (TEM) can provide more detailed information about structure and size (Lim et al., 2013[69]). To characterize the stability of colloidal disper-sions of NPs, zeta potential can be accessed by electrophoretic mobility. Usually, zeta po-tential of ± 20–30 mV is considered as an in-dicator of stability of colloids (Mourdikoudis et al., 2018[86]). For the detailed information about magnetic properties superconducting quantum interference device (SQUID) can be also used (Dulinska-Litewka et al., 2019[32]).

### Magnetic nanoparticles in GBM treatment

As for the current state of art there are many promising approaches that could be taken for the treatment of GBM using superparamagnetic NPs. Due to the location of GBM tumors, the NPs must have the ability to cross the blood-brain barrier (BBB) to act on the tumor site, which can be accomplished by coating with specific ligands for vascular targets (Gonzalez-Carter et al., 2020[50]). The targeting for the tumor can be more precise taking advantage of the paramagnetic properties of SPIONs by using an external magnetic field (Dulińska-Litewka et al., 2019[32]). The mechanism of action on the cells can be through delivery of existing drugs or it can rely on other properties such as SPION-mediated hyperthermia (Marekova et al., 2020[79]). Different studies involving magnetic NPs for GBM treatment can be seen in Table 1(Tab. 1) (References in Table 1: Babincová et al., 2018[12]; Fang et al., 2014[38]; Ganipineni et al., 2018[43], 2019[44]; Grillone et al., 2019[53]; Hradil et al., 2007[61]; Norouzi et al., 2020[90]; Pernal et al., 2017[92]; Prabhu et al., 2017[94]; Sun et al., 2016[113]; Zhu et al., 2012[129]).

#### Use in diagnostics

Magnetic resonance imaging (MRI) plays an essential role for radio therapy (RT) based GBM treatment regimens. Usually, MRI generates an image based on the decay of protons after magnetic pulses - characterized by T1 and T2 relaxation rates. Tumors tend to cause tissue changes that will alter these T1 and/or T2 rates, although these changes are usually hard to detect or to fully characterize without contrast agents that emphasize these changes. They are limited by their toxicity, rapid clearance, and need for a rapid administration. This makes development of non-toxic contrast agents that last longer in cells an interesting prospect (Charles-Edwards and De Souza, 2006[22]).

Recent studies have used gold NPs, as their low toxicity and ability to sensitize tumor cells to radiation therapy due to its high atomic number gives them potential as an anti-cancer agent. Gold NPs have shown to be able to enhance the effective radiation dose delivered to tumor cells by propagating electrons and free radicals induced by radiation, free radicals which will directly damage DNA and indirectly induce cell apoptosis (Sun et al., 2016[113]).

In an attempt to combine the imaging, diagnostic and therapeutic applications of iron and gold NPs, a formulation of gold and SPIO-loaded micelles (GSMs) coated with PEG-PCL polymer was studied by Sun and colleagues (Sun et al., 2016[113]). An *in vitro* model of GBM was used to investigate the radiosensitizing efficacy of the GSMs, in which they subjected cell lines to RT in the presence and absence of GSMs. They probed cells for gh2ax, a marker of dsDNA breaks, and calculated the density of *foci* in different treatment groups to evaluate whether GSMs would effectively potentiate radiation induced DNA damage.* In vivo*, they intravenously administered GSMs to mice implanted with human GBM tumors in either flank or brain and assessed micelle accumulation within these tumors. They observed that brain tumors exhibited less micelle uptake due to the blood-brain barrier. Finally, they used both CT and MRI on the mice with implanted tumors loaded with GSMs to evaluate the ability of GSMs to serve as contrast agents for imaging applications (Sun et al., 2016[113]). They found that GSMs were non-toxic at the dosages used, although they admit that further formalized toxicity studies must be performed to confirm their suitability. Despite this, the authors verified that GSMs were successful in effectively doubling the density of dsDNA breaks when compared to RT alone for both U251 and U373 cell lines, although it was unclear whether DNA break formation or inhibition of subsequent DNA repair were the primary driving force behind the discrepancy in DSBs in the two treatment groups, both of which described as mechanisms of gold NPs when used in radiosensitization (Sun et al., 2016[113]). Their results also showed that the administration and accumulation of a small amount of GSM in brain tumors would be sufficient to serve as a contrast agent for MRI-based visualization, and that CT imaging was not sufficiently sensitive to detect the gold portion of the GSMs. Furthermore, the persistence of GSMs in the tumor tissue for as long as 5 days after injection indicates that GSMs are durable enough to provide selective MRI contrast enhancement of tumor cells during treatment, in applications such as image-guided RT planning, monitoring of surgical resection, monitoring of response to other targeted therapies, and long-term imaging of tumor through the course of treatment progression (Sun et al., 2016[113]).

#### Hyperthermia-based approaches

Hyperthermia uses heat generated by magnetic NPs when they are subjected to alternating magnetic currents, inducing necrosis of the targeted cells and making the tumor more vulnerable to other coadjutant treatments such as chemotherapy and RT (Marekova et al., 2020[79]).

Hradil and collaborators developed dextran-coated ferric oxide NPs conjugated with specific anti-human epidermal growth factor receptor (HER2) aptamer and used them to induce magnetic hyperthermia in cultured cells (Hradil et al., 2007[61]). They started by synthesizing dextran-coated magnetic NPs and then conjugating the anti-HER2 aptamer with these NPs. *In vitro *tests were performed on human adenocarcinoma (SK-BR3) cell line, while as for the control for all experiments human GBM epithelial cells were used (U-87 MG cell line). Then, they proceeded to investigate the specificity of the NPs coated with the anti-HER2 aptamer through fluorescence activated cell sorting. Testing showed that the aptamer-tagged NPs were highly specific toward the HER2-expressing cells. In addition, a ninety-fold lower dose of the tagged NPs relative to that of the non-tagged NPs was needed to achieve ∼50 % cell death by hyperthermia of the SK-BR3 cell line, while for the U-87 MG cells, the viability level was close to 100 %. These results showed that targeted NPs can be applied at substantially lower doses than non-targeted ones to achieve similar effects of hyperthermia, which should greatly limit the side effects of treatment.

Pernal and colleagues explored a way of improving the effectiveness of magnetic hyperthermia (Pernal et al., 2017[92]). The authors showed that non-malignant cells, including human mesenchymal stem cells (MSCs) and primary mouse kidney and lung fibroblasts, displayed an unfavorably increased uptake of SPIONs when compared to brain cancer cells (E297 and U87). This means that when applying only SPIONs (with no other associated compounds) during treatment, the amount that ends up in the cancer cells is lower than the amount that goes to non-malignant cells, resulting in less effective treatment. To solve this problem, they use hydroxyapatite (HAP). Interspersion of SPIONs through HAP leads to stabilization of the maghemite and retention of strong magnetism even past that point, indicating an intimate interaction between HAP and SPIONs, reinforcing the proposed use of HAP as a chaperone-like carrier of SPIONs. The toxicity of SPIONs was also reduced following their embedment into a HAP matrix. Three SPION/HAP ratios were synthesized: 3.8 % weight percentage of SPION, 28.6 % weight percentage of SPION and only HAP. After the physicochemical characterization, they proceeded to cell viability assays, then a spheroid migration assay, a cytoskeletal anisotropy analysis and, finally, the magnetic hyperthermia analysis. They found that the HAP/SPION nanocomposites retained superparamagnetic properties, that they increased uptake in U-87 MG human GBM cells and healthy mesenchymal stem cells compared to SPIONs, and reduced the viability of brain cancer cells, even before magnetic hyperthermia. A functional synergy between the two components of the nanocomposites was established and as a result, the cancer *versus* healthy cell (U-87 MG/MSC) selectivity in terms of both the uptake and the toxicity was higher for the composite than for SPIONs or HAP alone, allowing it to be damaging to cancer cells and harmless to the healthy ones.

Babincová and collaborators use thermosensitive magnetoliposomes (MLs) co-loading SPIONs and doxorubicin and they tested against C6 rat glioma both *in vitro *and *in vivo *(Babincová et al., 2018[12]). They used a biocompatible colloidal suspension of citric acid stabilized magnetic NPs (fluid MAG-CT). Through heating experiments with an alternating magnetic field, they determined that formulations containing a higher concentration of fluid MAG-CT took less time to heat up and reach a temperature in the interval of 42-45 °C, necessary for cancer cell hyperthermia. The profile of drug release from the MLs was evaluated in the presence of an alternating magnetic field (AMF) and at physiological temperature (37 °C). Drug release from the MLs under AMF was 80 % after 10 min of exposure. Hyperthermia together with ML-based DOX drug delivery were evaluated in an assessment of cell viability. An *in vitro* hyperthermia experiment was done with MLs containing fluid MAG-CT NPs with or without doxorubicin, the values of relative cell number after hyperthermia treatment with fluid MAG-CT NPs was 79.2 %. For DOX-ML (with no hyperthermia treatment) the value was 47.4 %, and when both these modalities were combined the viability dropped to a minimum of 17.3 %.

#### SPIONs-based drug delivery systems

There are multiple approaches for the use of SPIONs in drug targeted delivery systems. Drugs can be ether encapsulated within SPIO structures directly or inside other types of NPs with SPIONs attached. Superoxide paramagnetic iron oxide nanoshells loaded with doxorubicin and curcumin prepared using hydrothermal and nano-precipitation method demonstrated 2-fold higher doxorubicin caspase-3 activity and 30-fold higher intracellular curcumin delivery (Zhu et al., 2012[129]). Its preferable accumulation at the acidic intracellular compartments such as endosomes and lysosomes enable pH-dependent drug release as well as nanoshells core dissolution for further reutilization of iron for in the body.

As for conjugated superparamagnetic NPs, there is an example of lactoferrin-tethered magnetic double emulsion nanocapsules (Lf-MDCs) to deliver hydrophobic and hydrophilic drugs simultaneously for more effective therapy and to overcome multidrug resistance (Fang et al., 2014[38]). Nutlins are a family of potent MDM2 antagonists that have shown to activate the p53 pathway (Villalonga-Planells et al., 2011[120]). Nutlin-3a and SPIONs were encapsulated in solid lipid NPs and showed high ability to cross BBB and had a higher pro-apoptotic activity *in vitro* on U-87 MG than the free drug (Grillone et al., 2019[53]).

Paclitaxel (Taxol^®^) is used as a drug for treatment of various types of cancer inducing a mitotic arrest in cells. The concentration of paclitaxel concentration in brain is not high enough for its therapeutic properties probably due to low ability to cross BBB and active efflux by ATP binding cassette transporters (Fellner et al., 2002[39]). It has been demonstrated that it is possible to increase paclixatel concentration in brain by nanoencapsulation. These NPs can be further loaded with SPIO (PTX/SPIO-NPs) to increase the accumulation of NPs threefold using magnetic targeting (Ganipineni et al., 2018[43]). These NPs can be grafted with RGD motif (PTX/SPIO-RGD-NPs) for active targeting of αvβ3 integrin. *In vivo *both NPs with magnetic and magnetic *plus *active targeting demonstrated a significant decrease of tumor volumes and a higher survival time compared to control groups (Ganipineni et al., 2019[44]). Doxorubicin is another drug used for treatment of multiple cancers and has low BBB permeability. *In vitro* studies performed on U251 cells demonstrated 2.8-fold uptake of doxorubicin when the drug was encapsulated in magnetic iron oxide nanoparticles stabilized with trimethoxysilylpropyl-ethylenediamine triacetic acid (DOX-EDT-IONP). The drug release could last 4 days and increased in acidic conditions. MDCK-MDR1-GBM co-culture model demonstrated the potential of achieving a higher BBB permeability of doxorubicin using DOX-EDT-IONP, and increased permeability and therapeutic effect with magnetic targeting and cadherin binding peptide (ADTC5) (Norouzi et al., 2020[90]).

Another strategy for targeting can be related to the use of specific antibodies against targets that are overexpressed in GBM cells in combination with superparamagnetic NPs. This approach was used for TMZ delivery using loaded polymeric nanocomposites conjugated with SPIONs, nestin antibody and transferrin or polysorbate-80 (Prabhu et al., 2017[94]). These nanocomposites were able to cross the BBB in orthotopic GBM xenograft model and could be targeted more precisely using a magnetic field.

## Liposomes

### Definition

Liposomes are vesicles typically sized between 50 nm to 100 nm made of minimum one phospholipid bilayer and an aqueous core. Liposomes are generally made of biocompatible, biodegradable materials, and are able to carry various types of drugs and biomolecules efficiently as water-soluble molecules can be carried within their aqueous core, whereas the lipid bilayer(s) encapsulate lipophilic/hydrophobic and amphiphilic molecules (Chamundeeswari et al., 2019[21]). Liposomes can be used for the treatment of different grades of brain tumors since they can cross the BBB through the inter-endothelial gaps of the highly vascularized, leaky BBTB in case of high grade brain tumors and transport across the intact BBB by means of receptor mediated transcytosis (RMT) or adsorptive mediated transcytosis (AMT) (Liu and Lu, 2012[70]). 

These nanosystems can be characterized by their physical and chemical parameters like their size, number of bilayers, surface charge and charge density, fluidity and presence of hydrophilic polymers and targeting ligands on the surface. In terms of the number of bilayers, liposomes can be classified as multilamellar vesicles (MLV) or unilamellar vesicles, with the second one dividing into large and small unilamellar vesicles (LUV and SUV). Bigger sized MLV are more adequate for encapsulating lipophilic or hydrophobic drugs than SUV due to the aqueous-lipid ratio (Dwivedi and Verma, 2013[34]).

A lot of success cases of liposome applications involve exploiting the enhanced permeability and retention (EPR) effect: these nanocarriers and drugs can be retained in tissues where vessels have defective endothelium and there is insufficient lymph drainage, and liposomes can fully exploit this effect (Maeda et al., 2001[77]). Tumors, once they reach a certain size (2-3 mm) start promoting angiogenesis to keep themselves supplied with oxygen and nutrients; however, the vessels thus created do not exhibit the same characteristics of normal blood vessels: their shape is irregular and their endothelium is leaky, thus creating this EPR effect (Iyer et al., 2006[62]).

### Production

**Figure 3:**
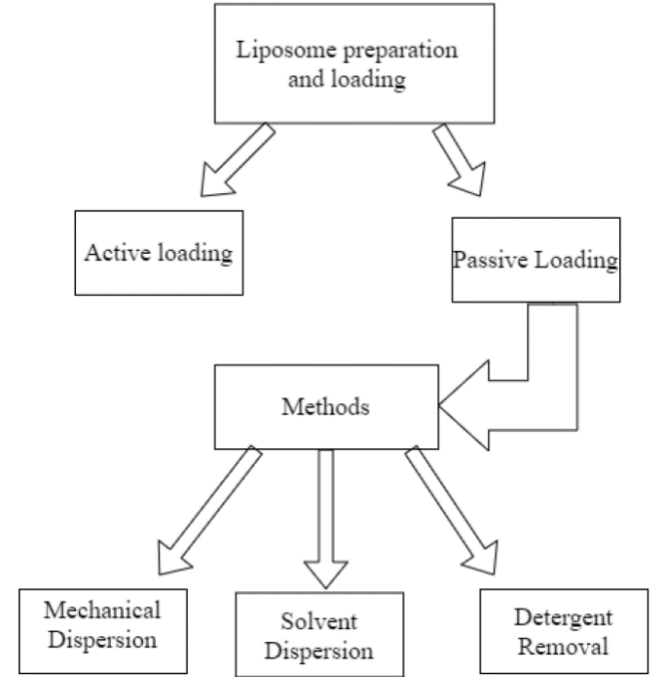
Common methods of liposome preparation and loading, adapted from Akbarzadeh et al. (2013).

All the methods for the preparation of liposomes include four basic stages: drying lipids from an organic solvent, redispersing them in an aqueous media, purifying the liposome that is formed and, finally, analyzing the product to detect any flaws in the process and guaranteeing its quality (Akbarzadeh et al., 2013[3]). Figure 3(Fig. 3) (Reference in Figure 3: Akbarzadeh et al., 2013[3]) summarizes common methods for liposome preparation and loading.

As shown, liposomes can either be actively or passively loaded, with passive loading happening during liposome production, and active loading being performed after the liposomes are already formed. A common active loading strategy involves filling liposomes with buffers or salt solutions inside, to load drugs that can only diffuse one way. By doing this, the drug alters its charge and is unable to cross the membrane, being trapped inside the liposome. Active methods like this tend to achieve higher, more stable encapsulation, though their higher stability also means they occasionally require active release methods to make the loaded drugs bioavailable (Gubernator, 2011[55]). 

Passive methods are varied, the most common method being the lipid film hydration method, a mechanical dispersion method which involves adding lipids to an organic solvent, mixing them thoroughly and then removing any excess solvent so that only the film is left. It is possible to do it with a stream of inert gases, or rotary evaporation for larger volumes. From here, buffer is added to hydrate the liposomes and mix with them, allowing the introduction of the drug to incorporate. It is typically followed by cycles of freeze-thaw creating the MLV. To limit the liposome's size, extrusion can be used, forcing the liposomes through filters and thus selecting the desired size (Xiang and Cao, 2021[126]).

### Current applications of liposomes

Liposomes are the oldest platform for nanomedicines and a widely researched topic when it comes to finding a formulation to treat several types of diseases including multiple forms of cancer, fungal infections, bacterial infections, immunosuppressants, pain relief, vaccines, and photodynamic therapy (Svenson, 2012[115]). Formulations currently in the market have improved on ubiquitous drugs, like doxorubicin, amphotericin B and even morphine, allowing for more controlled releases and better half-lives (Hafner et al., 2014[57]). There are several examples of success. 

In the early 90s, doxorubicin was encapsulated in liposomes, in the formulation known as DOXIL, or CAELYX. It was based on PEGylated liposomes used in a lot of cancers such as Kaposi's sarcoma, ovarian and breast cancer. This type of formulation led to an increase in drug half-life and its distribution through the tumor tissues. It also helps with the reduction of the cardiotoxicity associated with free doxorubicin. It has high stability when it comes to drug loading, through a gradient of ammonium sulfate, also allowing for its release in the tumor tissue and makes use of the EPR effect (Barenholz, 2012[15]). The great toxicity reduction is also a very important change from classical anthracyclines (Rafiyath et al., 2012[97]). It isn't, however, bulletproof, and multiple studies have appointed the major toxicity and adverse reaction being skin related events, mainly hand-foot syndrome (Lotem et al., 2000[74]). As for MYOCET, the non-pegylated version of doxil, it includes cyclophosphamide-loaded liposomes with about 150-250 nm. These liposomes also reduce the acute and chronic toxicity of the free drug (Bulbake et al., 2017[19]).

Another classic example is amphotericin B incorporated within liposomes, named AmBisome^®^. It is generally used for the treatment of serious and life-threatening fungal infections such as leishmaniasis, aspergillosis, blastomycosis and others. It is important to note that it interacts hydrophobically with the cholesterol components of the lipid membrane. These unilamellar bilayer liposomes have around 100 nm (Boswell et al., 1998[18]). 

More recently, lipid-based formulations used in the creation of COVID-19 vaccines were derived from liposomal technology to encapsulate nucleic acids in cationic lipid nanoparticles. Pfizer-BioNTech and Moderna's vaccines are built just like any other liposome formulation (Epling, 2021[35]). We need to consider what this means for nanotechnology, seen as when the world fought a crisis for 2 years, the answer was in nanoparticles lipidic formulations. In other words, if this is possible, maybe, with the urgency to find other treatment options for other diseases, nanotechnology can be looked at in another light (Germain et al., 2020[47]).

### Liposomes in GBM treatment

Standard drugs for GBM treatment are TMZ, bevacizumab, nitrosoureas such as carmustine, lomustine or nimustine, and tyrosine kinase inhibitors such as ibrutinib (Weller et al., 2013[125]; Wang et al., 2017[123]; Shergalis et al., 2018[103]).

None of these treatment options have approved as liposomal formulations yet, however many have attempted to create stable TMZ drug carriers. It has been attempted to encapsulate the drug and use PEG to protect the liposomes and allow them to reach the tumors. Still being in *in vitro* and *in vivo* studies, it has been shown to be very promising, since the concentration of TMZ reaching the tumor was greater and the distribution volume enhanced. Unfortunately, there were no significant changes in overall survival or tumor volume (Gao et al., 2015[45]; Nordling-David et al., 2017[89]).

There is another interesting project, with *in vivo* studies, that utilizes cisplatin in liposomal formulations to cross the BBB. This formulation proved to be effective in decreasing the toxicity of cisplatin to the overall organism and allowed for a better cellular uptake. However, it had a low encapsulation efficacy percentage, so if there was a chance to increase it, then perhaps this drug could find its way into clinical trials (Ashrafzadeh et al., 2020[10]).

Yoon et al. (2019[127]) attempted to revisit the concept of using itraconazole to stop the proliferation of cancer cells, differentiating them from regular cells. The main issue with this drug is the fact that itraconazole is a very insoluble drug, with a short half-life and low distribution volume, all of which can be fixed with the incorporation in liposomes, while also allowing for better targeting. In that study, the liposomes were prepared by the evaporation and film hydration method. It shows great promise for the future, seen as it successfully inhibited the *in vitro* cell proliferation.

Finally, as an additional example was done by Shi et al. (2018[104]) where they had four different liposomal formulations, prepared by reverse-phase evaporation method, changing between cationic and anionic and pegylation or no pegylation. Researchers found that carboplatin incorporated in any of these liposomes is a big advantage to treat GBM in comparison to free carboplatin. The cationic ones allow for a better attachment to target cells, while anionic and pegylated ones diffuse over a larger area of tumor.

## Polymeric Nanoparticles

Polymeric NPs are colloidal systems made up of natural or synthetic polymers that have a great potential for use in medicine, especially for gene/drug delivery (Martinho et al., 2011[81]). These NPs are within the size range from 1 to 1000 nm that can be loaded with active compounds, by trapping the compound inside the particle or adsorbing to the surface of the polymeric core (Neha et al., 2013[88]). The first polymeric NPs were made from non-biodegradable polymers, like poly(methyl methacrylate) (PMMA), polyacrylamide, polystyrene, and polyacrylates, but some toxicity issues occurred. Since then, biodegradable polymers, including synthetic polymers like poly(D,L-lactide) (PLA), poly(D,L-glycolide) (PLG), copolymer poly(lactide-co-glycolide) (PLGA), polyalkylcyanoacrylates, and poly-Ɛ-caprolactone, but also natural polymers such as chitosan, alginate, gelatin, and albumin, are preferred and more commonly used (Gagliardi et al., 2021[42]). 

**Figure 4:**
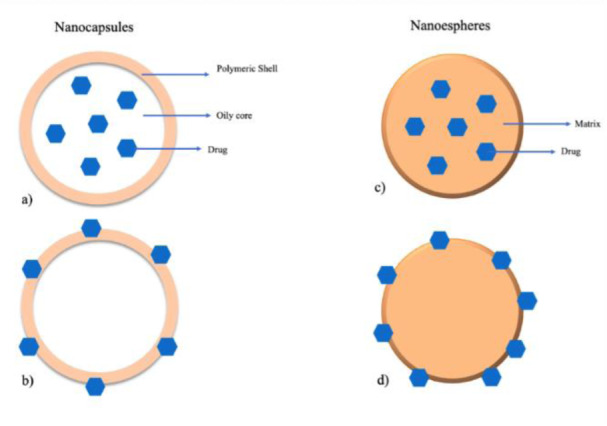
Schematic representation of the different polymeric NPs: (a, c) nanocapsules and nanospheres with entrapped drug, respectively; (b, d) nanocapsules and nanospheres with the drug adsorbed, respectively.

It is important to note that the term polymeric NPs comprises nanocapsules and nanospheres (Figure 4(Fig. 4)), which differ in their structure. Nanocapsules are reservoir systems which present a core in which the drug is usually dissolved surrounded by a polymeric shell, that controls the release profile of the drug from the core (Reis et al., 2006[100]) whereas nanospheres display a continuous polymeric network being classed as a matrix system. Here, the drug can be retained or adsorbed onto their surface. Rapamycin-loaded-polysorbate 80-coated PLGA nanospheres are an example of nanospheres presenting anti-glioma activity (Escalona-Rayo et al., 2019[36]). 

The potential use for controlled release of drugs, as well as the ability to protect drugs against the harsh environments, as well as the improvement of bioavailability and the therapeutic index of drugs, make some of the advantages of this type of nanosystem (Begines et al., 2020[17]; Mota et al., 2020[85]).

### Methods of polymeric NPs preparation

According to the intended applications and desired characteristics of polymeric NPs, or on the type of drug to be delivered, different preparation methods can be employed (Gagliardi et al., 2021[42]). Polymeric NPs are usually prepared from preformed polymers, in which toxicological and environmental hazard concerns may arise as organic solvents are usually required to dissolve the polymer, or by polymerization of monomers (Reis et al., 2006[100]; Gagliardi et al., 2021[42]). To minimize the mentioned concerns, solvent residues should be removed from the final product (Zielińska et al., 2020[130]). The most common methods used to prepare polymeric NPs are solvent evaporation, emulsification/solvent diffusion, salting-out and nanoprecipitation (Reis et al., 2006[100]; Nasir et al., 2015[87]; Gagliardi et al., 2021[42]).

The solvent evaporation method is one of the most used for the preparation of polymeric nanospheres preparation (Zielińska et al., 2020[130]). This method consists of an oil-in-water (o/w) emulsion, in which the organic phase is composed of an organic solvent, in which the intended drug and polymer are dissolved, and an aqueous phase which contains a surfactant (i.e., poloxamer 188, PVA, polysorbate 80 or other) (Sur et al., 2019[114]; Zielińska et al., 2020[130]). After the emulsification is formed by mixing organic and aqueous phases, the organic solvent is eliminated by evaporation. Different techniques can be used for solvent evaporation, such as continuous magnetic stirring at room temperature or in a slow process at reduced pressure (Sur et al., 2019[114]; Zielińska et al., 2020[130]). Then, the polymeric NPs can be washed and collected by centrifugation and stored (Zielińska et al., 2020[130]).

The emulsification/solvent diffusion method is also based on the formation of an o/w emulsion, but formed by a partially water-miscible solvent, containing the polymer and drug, in equilibrium with an immiscible aqueous solution with a surfactant (Piñón-Segundo et al., 2018[93]; Zielińska et al., 2020[130]). In similarity to the previous presented method, the emulsion is formed by mixing the organic and aqueous phases, and according to the oil-to-polymer ratio, the emulsification/solvent diffusion method can originate nanocages or nanospheres (Singh et al., 2018[109]). Despite requiring a high volume of the aqueous phase and the risk of diffusion of the hydrophilic drug into the aqueous phase, this is the most used method for the production of nanospheres (Zielińska et al., 2020[130]). 

Like the emulsification/solvent diffusion method, the salting-out method also relies on an emulsification resulting from the mixture of an organic and aqueous phase (Masood, 2016[82]). However, in the salting-out method, the aqueous phase contains the surfactant and a saturated salting-out agent, such as magnesium chloride (Masood, 2016[82]; Wang et al., 2016[124]). The organic phase, containing the polymer dissolved in an organic solvent (i.e., acetone), is emulsified in the aqueous phase by strong shearing forces (Wang et al., 2016[124]). To eliminate the salting-out agent, a final centrifugation step must be performed (Wang et al., 2016[124]). This method originates nanospheres that vary in size according to the polymer ratio used (Zielińska et al., 2020[130]).

Finally, nanoprecipitation, also known as the solvent displacement method, is mainly used for the encapsulation of lipophilic drugs (Martínez Rivas et al., 2017[80]). In this method, an organic solution, containing the drug and polymer, is added to an aqueous solution, containing a solubilizer, leading to the deposition of the polymer in the interface of the two solutions (Ahlawat et al., 2018[2]). For this, it is important that the polymer must be insoluble in the aqueous solution, in order to promote polymer deposition (Barreras-Urbina et al., 2016[16]). The NPs prepared following the nanoprecipitation method generally have a well-defined homogenous size (Zielińska et al., 2020[130]).

### Characterization of polymeric NPs 

Currently, there are no standards of NPs characterization approved or described by the Food and Drug Administration (FDA) (Crucho and Barros, 2017[29]). However, it is known that the properties and *in vivo* behavior of NPs depend on its physicochemical properties (Banik et al., 2016[14]; Crucho and Barros, 2017[29]). Regarding polymeric NPs, some physicochemical properties, that can vary, include concentration and composition, size and surface charge, morphology, crystallinity, and dispersion state (Zielińska et al., 2020[130]). In order to fully characterize polymeric NPs by evaluating some of the mentioned properties, an array of techniques is used, *i.e.,* electron microscopy, DLS, near-infrared spectroscopy, electrophoresis, and chromatography (Zielińska et al., 2020[130]). The importance of characterizing the physicochemical properties of NPs relies on the small variations in these parameters potentially resulting in detrimental changes in toxicity and therapeutic efficacy (Clogston et al., 2016[27]).

Different techniques can be used to measure the size of polymeric NPs, such as dynamic (DLS) and static light scattering (SLS), scanning and transmission electron microscopy (SEM and TEM, respectively), and atomic force microscopy (AFM), but the determined size varies depending on the method employed. Polymeric NPs' size can be affected by several factors such as the quali-quantitative composition and the amount of drug loaded, and this may lead to larger particles with broader size distributions (Zielińska et al., 2020[130]). Moreover, SEM, TEM and AFM also analyze the NPs' morphology, and AFM provides 3D information with high resolution at the nanometric scale (Zielińska et al., 2020[130]). The chemical composition of the atomic elements that make up an NP can be determined using an ensemble or single-particle elemental analysis method, including atomic absorption spectroscopy and time-of-flight mass spectrometry (TOFMS) (Sohail et al., 2020[111]; Zielińska et al., 2020[130]). After preparation, determining the molar mass distribution of the polymer provides information about the influence of the formulation components in the polymerization process, the occurrence of chemical reactions between the drug and the polymer. Size-exclusion chromatography (SEC) is the most used technique for this determination, besides that SLS is also used to analyze the intensity of light spread by the polymeric NPs (Zielińska et al., 2020[130]). The surface charge of the particles is reflected by the zeta potential (ζ) is very important as it enlightens how a polymeric NP interacts with the drug, behaves in biological fluids, and information regarding the formulations' colloidal stability. Doppler is one of the techniques used to determine particle velocity as a function of voltage, and this way the zeta potential is obtained from the electrophoretic mobility of particles in a respective solvent (Zielińska et al., 2020[130]). Moreover, drug-loaded polymeric NPs must also be evaluated regarding drug association and pharmaceutical release kinetics (Sohail et al., 2020[111]; Zielińska et al., 2020[130]).

### Dendrimers 

There is a very peculiar class of polymer-based nanosystems called dendrimers: synthetic polymers composed of branched units that emerge from a focal point, a central core, receiving their name by the similarity with a dendritic structure. Dendrimers may have various levels of branching, called 1^st^, 2^nd^ or 3^rd^ generations. These nanosystems have a sizable number of exposed anionic, neutral or cationic terminal functionalities on the surface, leading to hydrophilic or hydrophobic behaviour (Sohail et al., 2020[111]). Usually, dendrimers are composed of radially symmetric, globular, monodispersed and homogenous molecules, and present applications as delivery or carrier nanosystems for drugs and genes, although some dendrimers have intrinsically medicinal use, due to their antifungal, antibacterial and cytotoxic properties (Chis et al., 2020[25]). 

**Figure 5:**
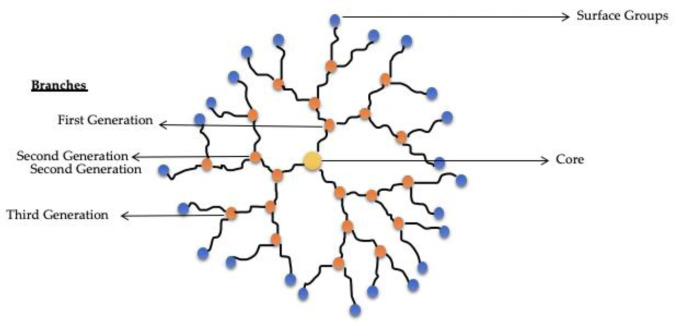
Schematic representation of a G3 dendrimer and its components.

Due to their single nanoscale properties and ability to enhance solubility, stability, oral bioavailability and drug targeting of various drugs, dendrimers, schematized in Figure 5(Fig. 5) have gained significant attention (Singh et al., 2019[110]). Poly(amidoamine) (PAMAM) dendrimers are one of the most commonly studied due to their availability through a robust synthesis and due to their features, such as mimicking peptide/protein (Sohail et al., 2020[111]). 

#### Production and characterization of dendrimers

These nanosystems are generally produced by a stepwise synthesis approach, giving them a controlled structure and narrow polydispersity. They can be synthesized by divergent or convergent approaches, or by a combination of both. In the divergent approach, by a series of repetitive reactions, dendrimers are formed from a multifunctional core that is extended outward. In the convergent strategy, through a series of inward-oriented reactions, small molecules start at the dendrimer surface and end up attached to a central core. A combined divergent/convergent approach combines the advantages of divergent and convergent synthesis (Sohail et al., 2020[111]). Recently, an advanced dendrimer synthesis emerged, by self-assembly of small dendritic components into large non-covalent supramolecular dendrimer (Sohail et al., 2020[111]). 

Assays commonly used to characterize dendrimers include high-performance liquid chromatography (HPLC), identifying and quantifying the separate components of a mixture or solution; mass spectrometry (MS), which measures the mass; charge ratio of ions; capillary electrophoresis (CE), that separate analytes by ionic mobility and are usually used to characterize low generation dendrimers; polyacrylamide gel electrophoresis (PAGE), that is a much cheaper alternative for separating nanoparticles based on their size and charge, having various advantages over CE; and small-angle neutron scattering (SANS), that measures small scattering angles to clarify the structure of substances with 1 to 100 nm. Each one of these methods has present advantages and limitations (Fana et al., 2020[37]).

### Polymeric-based NPs for the treatment of GBM 

Due to their size and other characteristics, NPs are being explored for the treatment of CNS tumors, such as GBM, being capable of carrying biomolecules, nucleic acids, and drugs, across the blood-brain barrier (BBB). This barrier plus the heterogeneity of brain tumors are the main reasons for unsuccessful treatments of this malignancy (van 't Root et al., 2017[118]). 

As demonstrated by other studies, it was observed that doxorubicin was able to reach the brain by loading doxorubicin in PLGA nanoparticles and then coating them with poloxamer 188 (Dox-PLGA). The Dox-PLGA nanoparticles produced a very notable anti-tumor effect against the intracranial 101.8 GMB rat model, representing a very good candidate as chemotherapy of brain malignancies that warrants more studies. The anti-tumoral efficacy of the optimized formulation and the ability of the NPs to penetrate into the intracranial malignancy and in the normal brain was then unconfirmed by the *in vivo* experiments (Maksimenko et al., 2019[78]). 

In another study (Agarwal et al., 2019[1]), enhanced uptake and amplified anti-cancer effects were observed in glioma cells due to receptor-based targeting of NPs. In this study, the authors confirmed a novel combined method for drug delivery to glioma cells. From the extensive *in vitro* studies, it was concluded that the synthesized NPs were highly safe and thus they will be shortly proceeding within* in vivo* biocompatibility and therapeutic efficacy studies of these NPs in the near future. More specifically, the biocompatibility and efficacy of both targeted and non-targeted nanoformulations with the drug morusin, an NF-kB inhibitor, as well as with other clinical drugs will be test. Hence through the results achieved, PLGA-MOR-CTX-NPs will be proposed as a promising nanoformulation for the development of future anti-glioma therapies. 

Another example includes TMZ. The use of PLGA NPs was proposed to improve the brain delivery of drugs used in GBM chemotherapy (Ramalho et al., 2018[98]). For that, NPs functionalized with an OX26 mAb for the transferrin receptor (TfR) were developed to target GBM cells, as these cells commonly present TfR overexpression. Stable NPs were prepared with suitable physicochemical properties for brain delivery, such as mean size smaller than 200 nm and negative charge. The developed NPs exhibited a good encapsulation efficiency of TMZ and they were able to maintain a controlled and sustained release of the drug for up to 20 days. Cytotoxicity studies showed that the encapsulation of the drug in PLGA NPs significantly improves the antiproliferative activity of TMZ. The use of the monoclonal antibody for TfR targeting proved to be advantageous in enhancing the cellular internalization of the NPs, suggesting that these are selectively uptaken by a transferrin receptor-mediated endocytosis mechanism in tumoral cells. Although the modification of the NPs with the OX26 mAb decreased the cytotoxic potential against GBM cells, the use of this antibody could enhance the permeability across the BBB *in vivo*, since BBB cells are also known to overexpress this receptor. As such, it is suggested that NPs functionalized with an OX26 mAb for TfR could be efficiently used for dual-targeting of both BBB and GBM cells. Future *in vivo *studies will allow evaluating the potential of the developed NPs for the treatment of GBM.

## Gold Nanoparticles

Colloidal gold, also known as gold NPs (AuNPs), is a suspension of nanoscale gold particles, with properties that differ from those of bulk gold. Most notably these NPs exhibit unique optical features. Depending on its size, AuNPs solution is either an intense red color (for particles smaller than 100 nm) or a dirty yellowish color (for larger particles) (Mody et al., 2010[83]). Similarly, the aggregation of AuNPs causes a color shift of the colloidal solution from red to blue, and their re-dispersion reverses the color change from blue to red (Ghasemi et al., 2018[48]). These intriguing optical properties are due to the NPs' unique interaction with light. Under the activation of light, conduction electrons on a noble metal collectively oscillate. This resonance, which often occurs on the metal surface, is known as surface plasmon resonance (SPR). When the resonance is restricted to nanoparticles it's called localized surface plasmon resonance (LSPR) (Mody et al., 2010[83]; Vines et al., 2019[121]; Bai et al., 2020[13]). When the LSPR occurs, the optical extinction of AuNPs can be maximized (more than 1000 times stronger compared to ordinary organic molecules), which strongly enhances the efficiency of photothermal conversion, photochemistry conversion and light energy absorption. If the absorption band of AuNPs is adjusted to the near-infrared region, these NPs can be used for photothermal therapy (PTT). Furthermore, AuNPs can serve as a contrast agent for surface-enhanced Raman scattering (SERS) and surface-enhanced fluorescence (SEF). Due to the high atomic number of gold, AuNPs have also been explored for radiotherapy sensitization for RT (Choi et al., 2020[26]). 

Again, the drug delivery to the brain is challenging because of the BBB, but due to the EPR effect it is already known that the accumulation of AuNPs is higher in tumoral tissue. Furthermore, as found by Choi et al. (2020[26]), the accumulation of AuNPs is higher in the brain hemisphere with GBM than without, probably due to GBM's disrupted BBB. AuNPs uptaken by GBM can also be further enhanced by administering NPs after RT, further disrupting the BBB. More traditional therapeutic opportunities include the conjugation of AuNPs with proteins, peptides, siRNA, and other drugs, to enable active targeting (Anselmo and Mitragotri, 2015[8]).

### Synthesis methods of AuNPs 

The most frequently used chemical method of AuNPs synthesis is the Seed-Growth method, which consists of reducing gold salts in the presence of a reducing agent, such as sodium borohydride. This originates tiny spherical seed NP. The next step involves the growth of these NPs in a solution containing metal salts and a weak reducing agent, like ascorbic acid. By modifying this procedure, it is possible to modify the morphology of NPs. This is commonly the case for the synthesis of gold nanorods, where a surfactant, commonly cetyltrimethylammonium bromide (CTAB), is used to allow the AuNPs to take on a rod-like shape (Amina and Guo, 2020[7]; Dumur et al., 2020[33]; Pellas et al., 2020[91]). Other chemical methods include the Turkevich method, the Brust method and digestive ripening (Amina and Guo, 2020[7]). The main disadvantage of a synthetic approach is that some chemicals used can be an environmental and/or health hazard. For this reason, various green methods using microorganisms like bacteria, fungi, plants and algae have been devised (Molnár et al., 2018[84]; Amina and Guo, 2020[7]) but also other methods with hazardous chemicals-free AuNPs for the treatment of melanoma (Silva et al., 2016[108]; Lopes et al., 2020[72], 2021[73]), breast cancer (Costa et al., 2020[28]) and thyroid cancer (Amaral et al., 2020[5], 2021[6]).

One key parameter for the synthesis of robust AuNPs with defined morphologies and functions is the choice of surface ligands. Ligands have a variety of functions, including regulating solubility and availability of active compounds during AuNP synthesis, minimizing the surface energy of NPs (necessary for colloidal stability), and encoding of NP functionality (Heuer-Jungemann et al., 2019[59]).

### Coating of AuNPs

Two parameters are fundamental for the design of new AuNPs: colloidal stability of the resulting solution, and easiness of functionalization. Another point to take into consideration is the possible desorption of the ligands (Dumur et al., 2020[33]). Furthermore, if we need to increase BBB permeation for brain delivery, NPs would benefit from a design that allows them to cross the BBB through transcytosis. For this, their surfaces can be modified, either non-covalently with a coating or covalently by functionalization (Heuer-Jungemann et al., 2019[59]). Nevertheless, the aggregation of AuNPs *in vivo* should be controlled. By coating AuNPs with a protective polymer and making them spherical or selenium-(Se-)terminated PEG, the stability of AuNPs in biological samples can be improved as well the aggregation can be avoided (Lu et al., 2021[76]). Herein, silica also appears to be an excellent coating candidate to prevent coalescence, due being chemically inert, optically transparent, and easily functionalizable (Dumur et al., 2020[33]). However, other issues related to silica *in vivo* uses are still on debate.

### Functionalization of the surface of AuNPs

The most direct way to functionalize AuNPs' surface is to synthesize the NPs in the presence of surface stabilizing ligands. In any case, functionalization of AuNPs surface seems to play an important role in the design of nanotherapeutic probes, controlling their pharmacokinetics, efficacy, and potential toxicity. Ligands that functionalize the surface of NPs should exert adequate colloidal stabilization and NP sealing from other molecules in challenging biological environments while functional ligands should increase NP targetability and perform distinct biological roles, also being responsive to external or internal stimuli (Silva et al., 2016[107]). 

**Figure 6:**
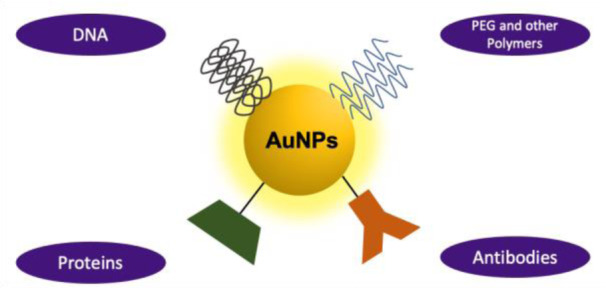
Surface modifications commonly seen in AuNPs for different applications.

To achieve maximum tumour penetration, NP size and characteristics must be precisely tuned in compliance with the tumor state. Increases in overall NP size, e.g., due to aggregation, could inhibit tumour targeting. Furthermore, very small NPs may leak into blood vessels, whereas very large NPs or aggregates of NPs may be cleared by macrophages and thereby fail to perform their therapeutic function. Therefore, selecting the appropriate ligand coating (Figure 6(Fig. 6) and Table 2(Tab. 2); References in Table 2: Cheng et al., 2014[23]; Dixit et al., 2015[31]; Gonzalez-Carter et al., 2019[51]; Gromnicova et al., 2013[54]; Guerrero et al., 2010[56]; Khongkow et al., 2019[64]; Lee et al., 2017[67]; Praça et al., 2018[95]; Prades et al., 2012[96]; Ruan et al., 2015[101]; Ruff et al., 2017[102]; Shilo et al., 2014[105]; Velasco-Aguirre et al., 2017[119]; Vio et al., 2018[122]) for the application is a critical issue (Heuer-Jungemann et al., 2019[59]; Hossen et al., 2019[60]; Lombardo et al., 2020[71]). 

#### Ethylene glycol containing ligands 

PEG is a special class of polymers with a wide range of molecular weights that have high solubility in water and a wide variety of organic solvents. PEG is generally used to stabilize NPs against aggregation, but also to inhibit the uptake by non-target organs, lengthen their circulating time in the blood, and improving NPs accumulation into targeted organs. 

PEGylation of NPs can be achieved through various routes. The simplest way is by adding PEG molecules during NP synthesis. 

PEG can play multiple roles simultaneously, acting as a solvent/cosolvent, a reducing agent as well as a capping agent. Since the PEG molecules are only loosely attached to the NPs, they can easily separate from the NP surface during processing steps (e.g., dilution/dialysis, centrifugation, heating, drying, aging, mixing with other compounds, etc.). 

PEG-modified nanomaterials tend to reduce immunological reactions, which can be attributed to the repelling nature of PEG to proteins. AuNPs functionalized with mPEG-thiol (5 or 10 kDa) is found to be susceptible to cysteine ligand displacement, resulting in increased serum protein adsorption. This displacement can be avoided by using alkyl moieties as hydrophobic spacers between the thiol and PEG. Although PEG has a low net charge, adding functional end groups like carboxyl or amine will result in a net negative or positive charge, which changes the properties of the PEG ligand and its interactions with biomolecules (Heuer-Jungemann et al., 2019[59]). 

#### Oligonucleotides

Oligonucleotides are another desirable ligands for NP functionalization due to their intrinsic properties of precise addressability and programmability (Heuer-Jungemann et al., 2019[59]). Generally, they display high target specificity, and ease of synthesis and functionalization. Via steric and electrostatic interactions, the DNA ligand shell stabilizes the NP nucleus, resulting in NPs that are extremely stable in several complex media. Electrostatic repulsion between adjacent DNA strands, as well as between DNA and the anionic AuNP surface, must be reduced to achieve high DNA loading on the NP surface. 

The anchoring group is another significant element that influences the stability of DNA-AuNP conjugates. As a result, di- and tri-thiol linkages, as well as bifunctional linkers like thiol *plus* amine, have been shown to provide higher conjugate stability than monothiols. 

#### Small peptides 

Peptide conjugation to NPs can result in increased reactivity due to a high local concentration, as well as multiplexing, which allows the use of the properties of multiple peptides at the same time. 

The grafting density of peptides on the surface of the NP, like that of oligonucleotides, must be closely regulated as it determines the overall stability, activity, and properties of the peptide - NP conjugate. It is also worth noting that, while a thick coating can improve NP stability, it can reduce peptide activity. Commonly used peptides for NP conjugation include cell-penetrating peptides (CPPs), which can enhance uptake and delivery of drugs across membranes and improve the efficiency of cellular uptake of nanoparticulate systems, as well as homing peptides, which are designed to target cells, tumors, and tumor-associated microenvironments. 

Peptides can be directly conjugated to the NP surface through free thiol-containing cysteine side chains in Au- or AgNPs. In addition to direct conjugation, peptides may be indirectly bound to ligands that present on the NP surface (Heuer-Jungemann et al., 2019[59]). 

#### Proteins 

Protein conjugation to NPs, like their smaller peptide equivalents, may be beneficial for a variety of reasons, involving an improved stability or possible self-assembly. As a result, peptide-NP conjugates have emerged as useful and promising methods for a variety of uses, including diagnosis and therapeutic approaches. The protein coating may be built to modulate NP stability, assess *in vivo* NP clearance, or target particular biological sites. 

Direct chemical covalent conjugation or electrostatic interactions can be used to functionalize NPs with proteins such as antibodies. If an incorrect approach and/or conjugation site is used, proteins can be harmed, unfold, and/or lose their structures. Proteins can be covalently conjugated to NPs, allowing for better regulation of protein activity as well as aggregation. Among the most commonly employed proteins for NP modification is avidin (Heuer-Jungemann et al., 2019[59]). 

The accurate synthesis and functionalization of inorganic NPs are critical for their colloidal stability and their performance. The type and nature of ligands determines NP toxicity, targeting capacity, drug distribution effectiveness, circulation in the body, association with proteins, cells, or more complex biological systems and, ultimately, the final application of the NPs. 

The rod- or branched-shaped AuNPs are a common form of AuNP used in PTT and drug delivery. Most of the time these particles are synthesized using toxic cationic surfactants like CTAB and hexadecyltrimethylammonium chromium (CTAC). Although several ligand exchange steps can be performed to remove those ligands before their use in biomedical applications, this is not cost and time-effective. 

Understanding the way that ligands conjugate to the NP surface, the strength of ligand−NP surface interaction, the net charge on the microenvironment around the NP, and how these characteristics change when NP size and morphology is varied are critical parameters to control the stability and function of NPs.

### Nanostructure of gold nanoparticles 

#### Shape 

**Figure 7:**
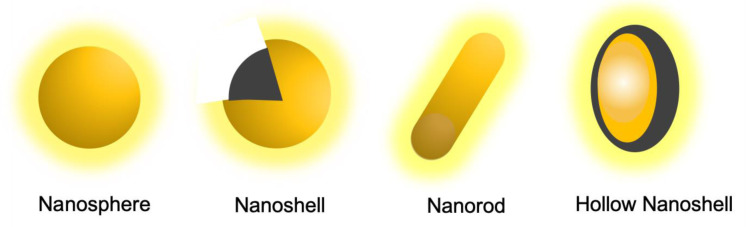
Different shapes of Gold Nanoparticles (AuNPs).

AuNPs unique optical properties can be changed by their size and shape (Figure 7(Fig. 7)). For example, spherical AuNPs of about 25 nm in size have ultraviolet (UV) absorption at 540 nm, and this absorption tends to red-shift as size increases. Additionally, if the absorption band is adjusted to the NIR, AuNPs can be used for PTT (Choi et al., 2020[26]).

##### Gold Nanorods (AuNRs) 

AuNRs are cylindrical AuNPs with typically less than 50 nm that are photothermally activatable. Due to their aspect ratio (length divided by width), they allow for the adjustment of the absorption band to the NIR region (650-1350 nm). Light in this wavelength range can penetrate more deeply into the human body due to the low absorption by tissue and blood. Thus, AuNRs have potential applications in PTT, differentiating them from other nanoprobes. However, their limitations with other high-resolution imaging techniques, like MRI, and irreproducibility in shapes led to the creation of nanoshells (AuNSs) (Mody et al., 2010[83]; Kaur et al., 2016[63]; Lombardo et al., 2020[71]). The last ones generally have shorter circulation times. Polarization of spheres into cylindrical structures requires a surfactant during synthesis.

##### Gold Nanoshells (AuNSs) 

AuNSs have a layer of gold surrounding a core made of dielectric material, most commonly silica due to its biocompatibility. Silica-gold nanoshells are usually 50-150 nm in diameter. This structure causes a red-shift of the absorption band of gold to the NIR region. This band is tunable by altering the core to shell ratio. In preclinical studies performed with these AuNPs, there was no indication of toxicity for durations of up to 404 days (Kaur et al., 2016[63]). 

##### Hollow Gold Nanoshells (HAuNSs) 

HAuNSs have a hollow core with a thin outer gold shell and a smaller size (< 100 nm) than silica-gold nanoshells while still presenting SPR in the NIR region. Their synthesis involves starting with a core-shell nanoparticle with a cobalt or silver core and a gold shell and then oxidizing the core to leave behind a hollow center. This hollow cavity can be used as a vehicle for drug delivery (Kaur et al., 2016[63]). Case in point, HAuNSs have been loaded with doxorubicin as a way to increase the efficacy of PTT ablation through combination with chemotherapy (Lee et al., 2013[68]). 

#### Size 

Previous work has shown that NPs between 40-60 nm are favorable for receptor-mediated endocytosis and have higher tumor accumulation rates than smaller NPs of 15 nm. In terms of tumor permeability, smaller NPs of 20 nm rapidly migrate throughout the tumoral tissues, whereas larger NPs primarily accumulate near vascular tissues. Furthermore, in smaller NPs renal clearance is facilitated (Feng et al., 2017[40]). 

#### Surface charge 

Neutral NPs are ideal for drug delivery due to their capacity to interact with cell membranes and to reduce immune response activation. Even though NPs with positive charge more easily interact with cell membranes, they also increase immune response, as well as the production of reactive oxygen species (ROS). It is described that positive charged NPs are rapidly recognized by the immune system. On the other hand, negatively charged NPs cannot interact so easily with cell membranes, therefore decreasing their internalization efficacy but they are more biocompatible (Grafals-Ruiz et al., 2020[52]).

### Current applications of gold nanoparticles

AuNPs currently present many applications in cancer therapy, such as PTT, RT and targeted delivery of small interfering RNA (siRNA) and microRNA, modulation cancers' gene expression.

PTT is a method that combines the use of a light source in order to increase the temperature of a superficial tissue, such as a superficial cancer (Amaral et al., 2021[6]). Its main goal is to cause thermal ablation of the tumor while minimizing damage to surrounding healthy tissue (de Paula et al., 2017[30]). Although promising, the effectiveness depends heavily on the deepness reached by the incident light and on the heat generated. For this purpose, NIR with higher tissue penetration capability, can be used to enhance the PTT effect. Biocompatible AuNPs designed to absorb light in the NIR, such as AuNRs and AuNSs, appear to be a worthy approach to improve PTT's efficacy and safety. When irradiated with light at their maximum absorbance wavelength, AuNPs locally convert the light energy into heat, which may lead to hyperthermia-mediated cell death of cancer cells, but non-targeted tissues remain unharmed (Rastinehad et al., 2019[99]; Costa et al., 2020[28]). 

RT consists of inducing DNA damage using both physically direct ionization and using free radicals by water ionization. The goal of RT is to deliver the maximum dose to the target tumor tissue while sparing surrounding normal tissue. Meaning, the maximum dose is determined by the toxicity to the surrounding healthy tissue. High-Z metal NPs, such as AuNPs, have been utilized to enhance the radiotherapy effect. Unlike the other radiosensitizers, that target specific biological pathways, high-Z metal NPs mainly enhance the physical dose delivered during radiotherapy by generating secondary X-rays, photoelectrons, and Auger electrons (Choi et al., 2020[26]). 

AuNPs targeting brain markers with glial fibrillary acidic protein can serve as a potential short interfering RNA (siRNA) nanocarrier. These AuNP-siRNA conjugates have been reported to knock down gene expression *in vitro* and *in vivo* (Glaser et al., 2017[49]; Yue et al., 2017[128]). MicroRNAs are a class of small non-coding RNAs that regulate diverse cellular processes through RNA interference-based mechanisms. Mature miRNAs are the endogenous equivalent of siRNAs. When siRNAs are incorporated into the RNA-induced silencing complex, they interact with, target mRNAs and inhibit them by translational repression or message cleavage (Silber et al., 2008[106]). 

Coated spherical AuNPs can carry siRNA at high surface densities. AuNP-siRNA conjugates are well protected from nuclease degradation unlike their free forms and provide highly efficient knockdown. This allows researchers to focus on designing NPs with a prolonged circulation time and tumor-specific targeting, instead of siRNA protection, thus accelerating development. To avoid GBM recurrence, the protein product of the delivered gene should be designed to be active in cancer stem cells (CSCs). The construct can also be under the control of a cancer-specific promoter, like survivin or PEG3, to ensure that healthy cells are not affected (Glaser et al., 2017[49]; Yue et al., 2017[128]). According to Silber et al. (2008[106]), targeted delivery of microRNA-124 and/or microRNA-137 to GBM tumor cells can inhibit proliferation of GBM cell lines, and thus could be valuable for the treatment of the disease. 

## Applications of Nanomedicine in GBM: Clinical Trials

There are different nanosystems and nanoparticles and some of the different types of nanosystems were as previously presented: superparamagnetic NPs, liposomes, polymeric NPs and AuNPs. 

To the best of our knowledge, there aren't many formulations of superparamagnetic NPs undergoing clinical trials for the treatment of GBM, and the GBM applications of these nanosystems are usually focused on inducing hyperthermia (Marekova et al., 2020[79]). One example of superparamagnetic NPs with such applications that have underwent clinical trials for GBM is NanoTherm^®^. These aminosilated-coated superparamagnetic iron oxide NPs led to an increase of the mean overall survival of GBM patients (from 6.2 to 13.4 months) (Chiarelli et al., 2015[24]; Aparicio-Blanco et al., 2020[9]).

Liposomes and polymeric NPs have also been extensively explored for drug delivery to GBM, and this is their main application in clinical trials (Grafals-Ruiz et al., 2020[52]). Examples of such liposomal formulations include liposomal Ara-C (DepoCyt) (NCT01044966) and liposomal irinotecan (NCT02022644). Similar to liposomes, polymeric NPs in GBM clinical trials are also focused on drug delivery of chemotherapy agents, with some examples presented in Table 2(Tab. 2). 

Another example is related to spherical nucleic acids (SNAs) where siRNA oligonucleotides arranged on the surface of a small spherical AuNPs can target the GBM oncogene BCL2L12. This nanosystem called NU-0129 (NCT03020017) is indicated for treatment of patients with recurrent GBM or gliosarcoma undergoing surgery. This oncogene BCL2L12 allows tumor cells to escape apoptosis, thus promoting tumor survival and growth. Intravenously administered SNAs showed evidence of crossing the BBB and reached patient tumors. The uptake of NU-0129 into glioma cells was correlated with a reduction in tumor-associated Bcl2L12 protein expression. No significant treatment-related toxicities were seen. Severe (i.e. higher than grade 3) adverse events were observed in two patients, which were considered as “possibly related”. Macrodosing of the nanotherapeutic NU-0129 was well tolerated in GBM patients (Kumthekar et al., 2021[66]).

Table 3(Tab. 3) summarizes some registered clinical trials of nano-based systems for the treatment of GBM.

## Conclusion

GBM is a very aggressive type of brain cancer that has very poor treatment outcomes, due to inoperability, the BBB limiting drug crossing, and therapy resistance. 

For all previous reasons, this malignancy remains virtually uncurable. This review explores the applications of organic and inorganic nanosystems in treatment and diagnosis of GBM. 

Research on the application of nanomedicine to GBM treatment has been blooming, with several publications but also ongoing clinical trials with very promising results.

Selectively increasing the permeability of BBB to particular drugs, increasing the sensitivity of the tumor cells to PTT and hyperthermia, and increasing the specificity of the treatment to malignant tissue are examples of approaches with promising achievements. In this review, the most representative examples (not all) on how different nanosystems were employed to improve existent treatment methods, or overcome challenges in treatment. It is suggestive that the application of the strategies reviewed achieve success in GBM treatment beyond preclinical and clinical trials in the near future as well. That said, expectations must be tempered: even with all the research conducted in GBM treatment, and with all the nanosystem based solutions proposed to tackle this disease, if we are to look back into past research and clinical trials, we will see that no nanosystem to date, even those that have achieved preclinical and clinical success, has been able to increase overall survival enough to supplant the current standard of care. However, this may yet change or will certainly change in a very near future.

## Notes

Mariana Amaral and Nuno Cruz contributed equally as first author.

## Declaration

### Funding

This research was funded by Fundação para a Ciência e Tecnologia (FCT): Project Reference UIDB/04138/2020 and UIDP/04138/ 2020 but also the PhD fellowships SFRH/BD/ 05377/2021 and UI/BD/150754/2020.

### Conflict of interest

The authors declare no conflict of interest.

## Figures and Tables

**Table 1:**
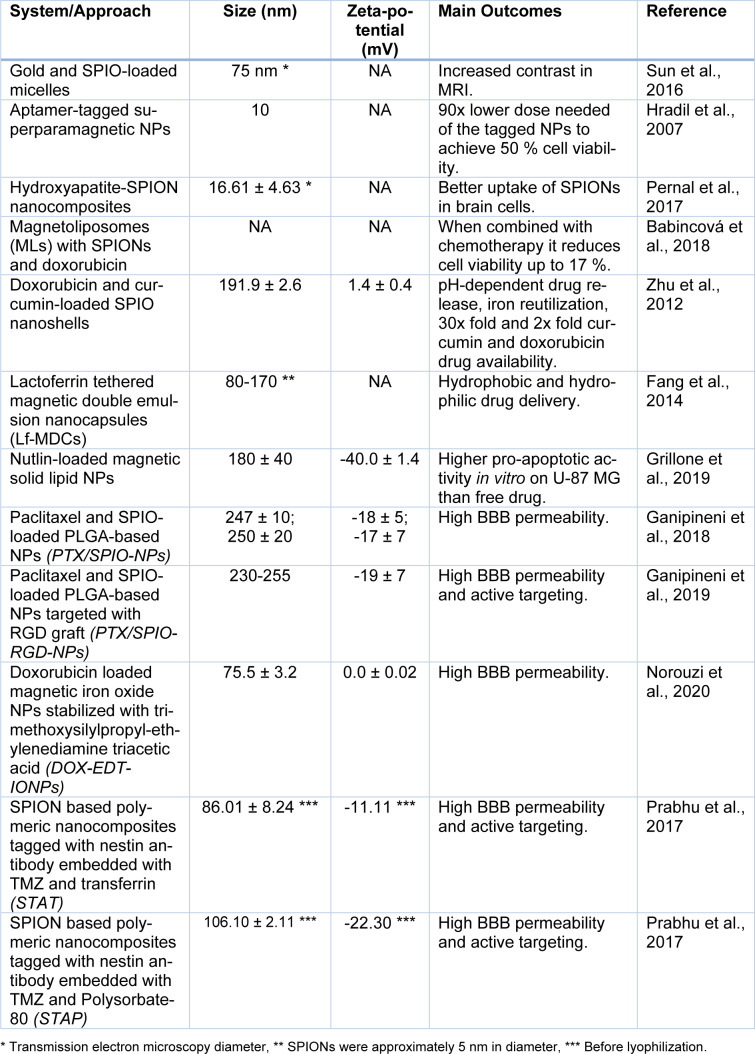
Applications of paramagnetic NPs in treatment of GBM.

**Table 2:**
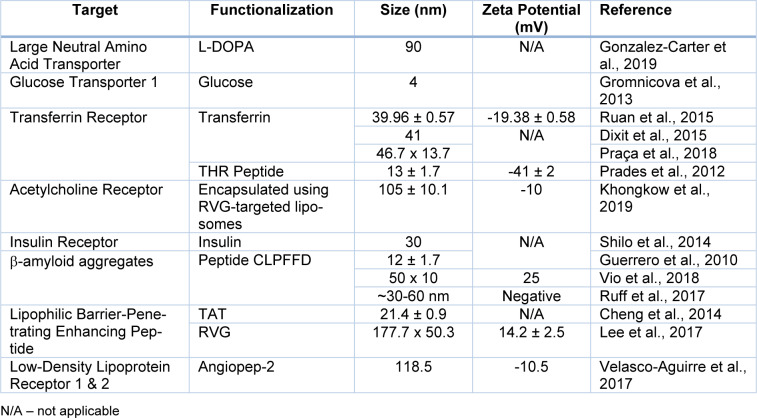
Summary of possible surface functionalization strategies for improved BBB crossing of Gold Nanoparticles (AuNPs).

**Table 3:**
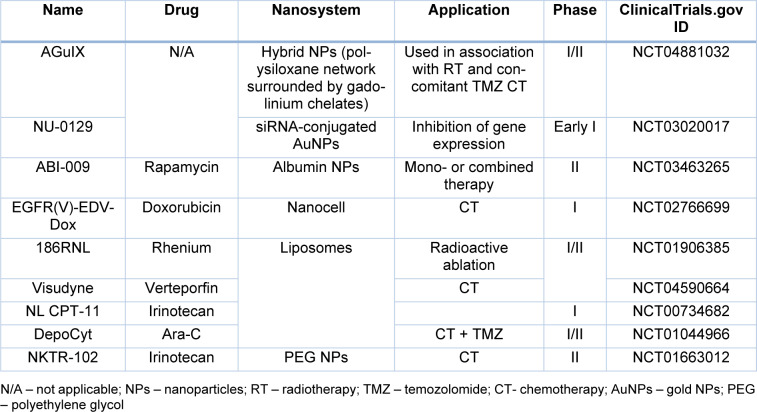
Clinical Trials registered in ClinicalTrials.gov using Nanosystems for the treatment of GBM.
